# Humans Predict Action using Grammar-like Structures

**DOI:** 10.1038/s41598-020-60923-5

**Published:** 2020-03-04

**Authors:** F. Wörgötter, F. Ziaeetabar, S. Pfeiffer, O. Kaya, T. Kulvicius, M. Tamosiunaite

**Affiliations:** 1 Universität Göttingen, Department for Computational Neuroscience at the Bernstein Center Göttingen, Inst. of Physics 3 and Leibniz Science Campus for Primate Cognition, Göttingen, Germany; 2 Vytautas Magnus University, Faculty of Informatics, Kaunas, Lithuania

**Keywords:** Human behaviour, Engineering

## Abstract

Efficient action prediction is of central importance for the fluent workflow between humans and equally so for human-robot interaction. To achieve prediction, actions can be algorithmically encoded by a series of events, where every event corresponds to a change in a (static or dynamic) relation between some of the objects in the scene. These structures are similar to a context-free grammar and, importantly, within this framework the actual objects are irrelevant for prediction, only their relational changes matter. Manipulation actions and others can be uniquely encoded this way. Using a virtual reality setup and testing several different manipulation actions, here we show that humans predict actions in an event-based manner following the sequence of relational changes. Testing this with chained actions, we measure the percentage predictive temporal gain for humans and compare it to action-chains performed by robots showing that the gain is approximately equal. Event-based and, thus, object independent action recognition and prediction may be important for cognitively deducing properties of unknown objects seen in action, helping to address bootstrapping of object knowledge especially in infants.

## Introduction

Experienced craftsmen will intuitively understand each other’s actions even without words and their interaction is usually efficient and fluent. This is because cooperation between humans is heavily based on fast, mutual action prediction, originating already in non-human primates^1^. When we observe someone else, we can understand his or her actions before completion and even without any object present, like in the case of a pantomime. This allows us to perform our own actions early; seamlessly blending different action streams together similar to a dance. Currently existing artificial systems, on the other hand, are still not very good at action prediction. The fact that the same action can exist in so many variants, for example with different trajectories, objects, and object orientations (poses), makes machine prediction of action a hard problem.

Several methods have been employed to address this problem. Actions are most of the time analyzed using video data^2–4^, frequently based also on the objects that take part in the action^5–8^ and sometimes supported by an analysis of object affordances^9^. Alternatively, emphasis can also be laid on the spatio-temporal structure of the actions^3,10^ or on predictive models^11,12^. Plan recognition is one other possible approach to analyze and predict actions^13–16^. Some approaches apply action grammars, which can be used to aid recognition and prediction^17–20^. Current deep learning based approaches implicitly use most of the aspects mentioned above^21–24^. Thus, the diversity and complexity of technical methods for action recognition and prediction is large and one can ask why it seems so effortless for humans to do this? For example, even a two-year old child will easily and quickly distinguish between her mother making a sandwich or doing the dishes. Children (and – of course – healthy adults) do not get lost in the details of the actual action execution. Somehow, we manage to extract the action’s essence from which we quickly arrive at its semantics allowing us to predict it early.

During the last decade, others and we have tried to address action recognition from a more abstract, grammatical perspective. The essence of a sentence is in most languages given by the word order, where “Is there a cat?” is a question while “There is a cat.” is a statement. This difference will prevail if we replace “cat” with “dog” and/or “there” with “this”. Hence, the grammatical structure is, on its own (without considering specific words), a strong indicator of the basic meaning of a sentence. The analogy between actions and sentences is striking. Actions can fundamentally be distinguished by the sequence-order of the spatial and temporal relations between the different objects – including the hand – during the action^25–28^. This sequencing could be considered as the action’s grammar^17,20,29,30^, where objects take the role of words.

**Figure 1:**
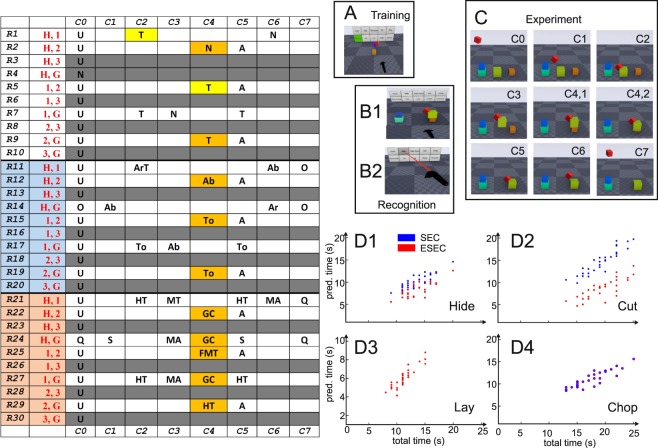
Left: Extended Semantic Event Chain (ESEC) of a “Hide” Action. Right: Experimental design (**A**–**C**) and relation between prediction time and total time of four different actions for all their 30 action videos (**D**). Abbreviations in the ESEC are: T: touching, N: non-touching, A: absent, O: very far (static), Q: very far (dynamic), Ab: above, To: top, ArT: around with touch, S: stable, HT: halt together, MT: move together, FMT: fixed-moving together, MA: moving apart, GC: getting close. Note that the leftmost column, C0, is the same for all actions and indicates the start situation before any action.

Recently we introduced the framework of Extended Semantic Event Chains (ESEC), which is a highly compressed tabular action representation (Fig. 1*, left*), encoding along its columns only the *temporal sequence of changes of object-object relations* in an action. This framework can be used to automatically recognize observed actions, which is achieved in the following way: Action start and end are well defined by the fact that the hand is at start *still* free (or at the end *again* free) not touching anything. ESECs then encode actions in three sub-tables (Fig. 1*, white, blue, orange colored rows, see left side*). The white part of the ESEC encodes changes in touching (T) and un-touching (N) events between the objects, the blue part their static spatial relations (e.g., *Ab*: One object is *above* another object, etc.), and the orange part dynamic ones (*GC*: One object *gets closer* to another object, etc.).

We define the following types of static spatial relations: “Above” (**Ab**), “Below” (**Be**), “Right” (**R**), “Left” (**L**), “Front” (**F**), “Back” (**Ba**), “Inside” (**In**), “Surround” (**Sa**) and “Between” (**Bw**) and “Around” (**AR**) and the following dynamic spatial relations: “Moving Together” (**MT**), “Halting Together” (**HT**), “Fixed-Moving Together” (**FMT**), “Getting Close” (**GC**), “Moving Apart” (**MA**) and “Stable” (**S**). The exact definitions for determining all these relations are given in the supplementary material.

Interestingly, we found that five types of objects will always suffice to describe any possible action: Hand, H; Ground, G; and *abstract* objects 1, 2, and 3. The latter are numbered by the occurrence of the corresponding *real* objects during an action. Object 1 is that object, which is first touched by the hand, Object 2 is the one first un-touched by Object 1, and Object 3 is the one first touched by Object 1. It is impossible to create single-handed actions that contain more objects^31^, but relations can exist between any of those five entities leading to ten rows in each sub-table. Note that in some actions, like the “Hide” action shown in Fig. 1, not all objects play a role, resulting in empty (gray) rows in the table. Gray rows also occur if an object-object relation never changes during the action. Furthermore, note that objects “come into being” only at the moment when they are touched (or un-touched), before that they are “undefined” (U). See Suppl. Material for the definition of all the different, here-used, relations, the geometrical assumptions to determine them, and their thresholds.

Using this encoding, here we show an ESEC of a “Hide” action (Fig. 1*, left*), where one object is moved over another one to cover it. This action was simulated in virtual reality (VR) using cube-like “objects” to avoid that the observer would be able to recognize the action just by looking at the objects. The same VR-method has been used to create the results of this paper and methodological details will be given below. Here we first show that panels C1 to C7 in Fig. 1*, C* capture the seven characteristic change-moments that happen during this action, which correspond to columns C1 to C7 in the ESEC. For example, C2 represents the moment when the hand-object (red cube) touches object 1 (the green cube). Thus, the whole, complex action can be reduced by this framework to seven states only.

Using the ESEC encoding, it is possible to distinguish at least 35 different single-handed manipulation actions and we had argued that humans might not have many more available in their repertoire^31^. It is furthermore important to note that all these actions are recognized early. On average only 56% of the action duration needs to have passed before an action will be known^31^. For the Hide action, this happens just a bit later, at column C4.

In the current study we fundamentally ask, whether humans – in the absence of object knowledge – would potentially use the same, underlying “algorithm” of analyzing object-object relations for action recognition and prediction. This is an important question as it specifically concerns the bootstrapping of action and object knowledge in very young children. The realm of (human made) objects is vast and it is a non-trivial cognitive problem for a child to understand their meaning. While 35 found manipulations might not capture all, this is, on the other hand, indicative of the fact that the number of actions is far more limited than that of objects. Would it not help if a child were able to understand actions without objects? For example, understanding the “essence of a cutting action” would allow bootstrapping object knowledge for various – never before seen – cutting tools. The fact that children, during their play, very often completely redefine and abuse things according to their action plan points to the primary role of action and lets these things become the action’s objects, regardless of their adult meaning and common use (see OAC concept^32,33^).

In the following, we present the results from a series of virtual reality-based experiments using moving colored blocks (Fig. 1A–C) instead of real objects asking our participants to indicate at what moment they would recognize the one action shown out of ten possible actions. The central novel contribution of this study is the finding that humans recognize the majority of these ten actions indeed at the same event column as the ESEC, pointing to the strong influence of object-object relations in action recognition. This may prove useful for seamless human-robot interaction and we also show how two different robots substantially gain performance speed when chaining actions in such a predictive manner.

## Results

### Quantitative analyzes

Panels D in Fig. 1 plot the prediction times against the total times for four different VR-action types. Plots show results for ESECs (red) as well as SECs (blue), where in the latter case only the top sub-table (touching/non-touching) of the ESEC was used. This corresponds to the original SECs as introduced in our older studies^25^. In general, all relations are linear. Note that SECs cannot distinguish between several actions. For example, Lay is confused with Shake and therefore in panel D3 only ESEC performance is shown. For Chop (D4), ESECs and SECs are equally predictive. The linear relations seen here holds for all actions and this allows us to define a measure called “predictive power ***P***” (see Methods section, below), needed to analyze human results and to compare them to ESEC performance, by relating prediction time to total action time. The ***P***-measure essentially quantifies “how fast” someone will predict a certain action. For example, a ***P***-value of 50 corresponds to action prediction happing after half of the action had been performed and ***P*** = 0 means that the action had to be completed before it was recognized.

**Figure 2:**
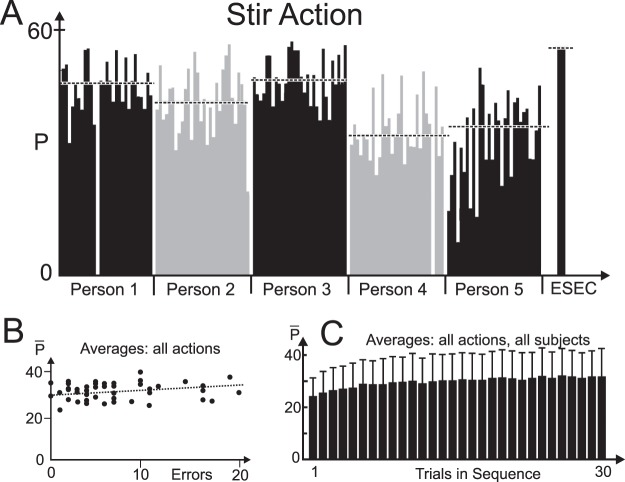
(**A**) Individual performance, plotting predictive power *P* for all 30 trials of the Stir action of five subjects and the ESEC (median: dashed). (**B**) Predictive power averages $\overline{{\boldsymbol{P}}}$ over all actions plotted against the number of recognition errors for all 300 trials for 49 subjects (dots). (**C**) Predictive power averages $\overline{{\boldsymbol{P}}}$ and std. dev. over all actions and all subjects plotted against trials.

Figure 2A shows values for ***P*** for five subjects demonstrating their performance for the Stir action. Some variability exists and white gaps are cases where no or wrong recognition has taken place. Subject 5 possibly shows small and gradual improvement over the first 10 trials indicative of a small degree of learning (see discussion of Fig. 2C, below).

Subjects had been prompted to perform immediate button-press on recognition but without any time-competitive recognition-speed requirements. We avoided this to create a more realistic query situation potentially related to ‘natural’ action prediction in daily life (which usually happens also without the need of doing this extra fast). Therefore, we observed only a small range of average predictive power values $\overline{{\boldsymbol{P}}}$ across our 49 participants (Fig. 2B). Interestingly, there is only a very small trend that faster participants, that have higher $\overline{{\boldsymbol{P}}}$ values, produce more recognition errors than the slower ones.

We also asked to what degree learning takes place along the trials as suggested by the one case discussed above. The grand average $\overline{{\boldsymbol{P}}}$ across all actions and subjects indeed shows a very small degree of increase for the first trials (Fig. 2C), which is so small that in respect to the standard deviations that we could ignore it for the remainder of this study.

**Figure 3:**
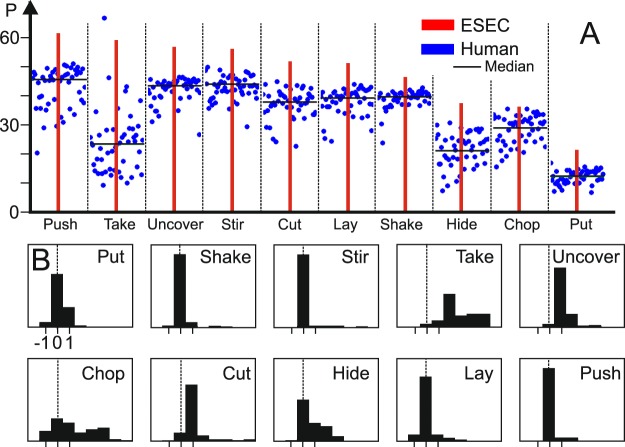
(**A**) Predictive power of ESECs (bars) and of 49 humans (dots) for all 30 trials of ten actions rank ordered according to ESEC performance. (**B**) Histograms of the frequency of human prediction in the same column than the ESEC (relative column number: 0, dashed line) or earlier/later (=left/right of dashed line). Total number (integral) in each histogram is about 1500 (exactly: 49 × 30 minus false recognitions).

Figure 3A shows how ESECs and humans differ in respect to predictive power for the ten different actions. In general, humans predict later than ESECs, where only for Take Down (“Take” in the figure) a strong difference exists. The most interesting question, however, is to ask at which event-column humans predict the actions. In Fig. 3B we plot how often humans predict an action using the same event column (x-axis label: “0”) as that where prediction happens in an ESEC, or earlier (x-axis: negative numbers) or later (positive numbers). With the exception of Chop and Take Down most distributions are narrow and – indeed – for six out of ten actions human prediction clearly happens most often at the same column as for the ESEC.

Table 1 shows a summary statistics for the results in Fig. 3B. Many times the histograms in the Figure are highly skewed. Therefore the median is a better indicator as compared to the mean. Here we find – corresponding to prima vista analysis of the histograms – that six actions have a median of zero and two a median of one. The columns “Zeros” and “Ones” give the percentage of zero and one-entries in the histograms. Confidence intervals of the median are narrow and often collapse to a single value. This confirms the above-made observations that in six cases humans very likely use the same event as that used by the ESEC algorithm, whereas in two cases (Cut and Uncover) the next-following event is more indicative. Only Take and Chop do not fit to this picture.

**Table 1:** Summary statistics for Fig. 3B.

	chop	cut	hide	lay	push	put	shake	stir	take	uncover
Skewness	0.36	2.62**	0.77*	1.99**	3.30**	0.039	4.84**	5.48**	0.038	2.10**
Zeros (%)	20.70	11.27	51.42	79.78	90.55	66.71	90.72	93.57	2.70	4.12
Ones (%)	23.28	72.18	22.55	7.43	4.79	25.63	5.15	2.29	8.02	75.86
Median	2	1	0	0	0	0	0	0	2	1
95% Conf. Int. Median	[1, 1]	[1, 1]	[0, 1]	[0, 0]	[0, 0]	[0, 0]	[0, 0]	[0, 0]	[2, 3]	[1, 1]

The asterisks refer to *=medium degree, and **=high degree of skewness.

The fact that the predictive power of humans (Fig. 3A) is lower than that of the ESECs is, very likely, owed to the fact that ESEC instantly predict when the relevant event happens, whereas humans probably take a bit more time to accumulate more evidence about the action.

### Robot example experiments and human-based performance gain quantification

To demonstrate the temporal gain that good (versus less good) action prediction can achieve, we used two KUKA LWR robots equipped with Schunk 3-finger hands and let them “play an action-sequence speed game”. The target of this game was to perform as fast as possible five different actions on a set of hollow cubes; two by the one and three by the other robot. All five actions came from the action set used in the VR experiments and were “put on top”, “hide”, “shake”, “take down”, and “push”. To play the game, robot 1 had, at the beginning, to perform one freely chosen action, robot 2 had to recognize as fast as possible what robots 1 does and then perform another from the remaining four actions. Next robot 1 needed to find one of the remaining three actions, etc., until all actions were done.

The choice of these specific five actions came from the analysis of human actions using the VR action videos, created by human demonstration. Hence, these videos reflect realistic human action performance. From the existing ten actions, we selected the actions “put on top”, “hide”, “shake”, “take down”, and “push” – as mentioned above – because they cover different situations: early versus late prediction times as well as large versus small differences between ESEC and SEC prediction times including one action, where SEC and ESEC prediction times do not differ. Average timings, on which this selection was based, for these actions performed by humans are shown in Table 2. Note that all actions had been taken from the action videos used in the VR display. For all the videos there is a unique mapping of every ESEC column to the actual moment in time when this column happens, because we know exactly at which movie frame a certain ESEC-event occurs. This allows calculating all time-values (given in seconds) that are mentioned in the following tables and figures.

**Table 2:** Average performance measured from human demonstration used to create the VR experiments.

	Average Action Duration [s]	Average ESEC Prediction Moment [s]	Average SEC Prediction Moment [s]
Take Down	11.7 (2.9)	3.3 (0.7)	3.3 (0.7)
Put on Top	12.0 (2.1)	8.0 (1.9)	9.2 (1.7)
Shake	12.5 (2.1)	6.5 (1.2)	10.8 (1.7)
Push	12.7 (1.9)	5.0 (1.1)	10.0 (1.6)
Hide	13.8 (2.5)	8.3 (1.6)	10.3 (1.5)

Numbers in brackets are standard deviations.

In the robotic experiments, we aim to compare action predictions using ESECs against those when using only SEC (touching/non-touching) information, where the baseline is the sequencing of those five actions without any predictive mechanism.

**Figure 4:**
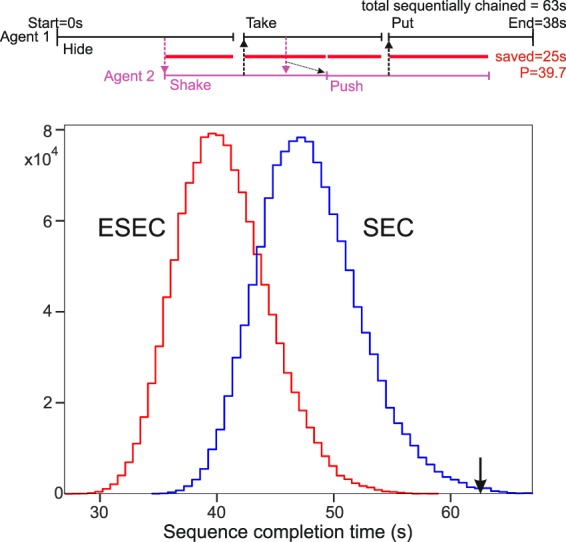
Top: Example of a sequence “Hide-Shake-Take down-Push-Put on top” using ESEC predictions and human action execution times (black and pink lines) for 2 actors. Dashed arrows show prediction moments and red lines the temporal savings. Bottom: Distribution of sequence completion times of humans based on 1.2 million combinations of the five actions as taken from the VR-actions setup. Distributions are significantly different (paired t-test, p<<0.000001). The arrow marks the average human sequence completion time without action interleaving of 62.6 s (std = 5.10, paired t-test against sequence completion time using ESEC or SEC, p<<0.000001 in both cases).

The top part of Fig. 4 shows a timing diagram of one possible sequence “Hide-Shake-Take down-Push-Put” with timings (to scale) taken from five individual human VR actions and predictions based on ESECs. Agent 1 starts with the hiding action and at 8.5 s Agent 2 can predict this (leftmost downward arrow) and start its own shaking action and so on. The leftmost red horizontal bar shows how many seconds have been saved by this prediction. In the end, this adds up to 25 s of savings from a total non-interleaved chain length of 63 s. This results in a ***P*** value of 39.7. The timing diagram also shows the complexity that any such 5-action chain may express. Often there are waiting gaps existing or an agent cannot yet make use of a prediction, because its own action has not yet finished (indicated by the slanted, dotted arrow).

In Fig. 4, at the bottom, we show the distribution of human action sequence completion times that would arise when either using ESEC-based or SEC-based prediction for 1.2 million possible sequence combinations of the five different actions (for details see Supp. Material) automatically creating and evaluating their timing diagrams. The total action sequence time without prediction is for humans on average 62.6 seconds (Table 3).

**Table 3:** Performance of ESEC and SEC for human-based data and for robots for different sequencings of five actions and different prediction mechanisms.

		Sequence Execution Time [s] and P values							
		HUMAN-based				ROBOT			
		Total without prediction		Avg.: 62.6 (5.1)		Total without prediction		1	75.2
								2	83.1
								3	75.9
	**Action Sequence**								
		**Human ESEC**	***P***	**Human SEC**	***P***	**Robot ESEC**	***P***	**Robot SEC**	***P***
1	Take, Hide, Shake, Push, Put	37.8 (3.6)	*39.6*	47.0 (3.7)	*24.9*	44.3	*41.1*	52.7	*29.9*
2	Push, Put, Shake, Hide, Take	40.5 (3.9)	*35.3*	51.9 (4.3)	*17.1*	57.5	*30.8*	62.9	*24.3*
3	Put, Shake, Take, Hide, Push	42.1 (3.7)	*32.7*	47.0 (3.6)	*24.9*	47.0	*38.1*	50.9	*22.9*
Average P:			***35.9***		***22.3***		***36.7***		***25.7***

The small font indicates that human – very likely – will not use a SEC-based prediction mechanism. Differences between ESEC and SEC sequence execution times for the human-based data are significantly different and also against the total time without prediction (paired t-tests, p ≪ 0.000001).

Note that humans (as shown above, Fig. 3) seem to base their predictions on ESEC-related judgement. Thus, SEC-based sequencing does not occur in reality and we show the SEC-based distribution (Fig. 4) and numbers (Table 3) only for comparison purposes.

The ESEC histogram demonstrates that action sequences would be completed within less than 30 up to more than 55 seconds where the actual numbers depend on the scene geometry. Also – as expected – the ESEC-histogram is centered at shorter times than the SEC-histogram, a fact that is also reflected by the averages in Table 2.

For the robotic experiments, three example sequences (Table 3*, numbered 1 to 3*) of different sequencings, ordered by their human-ESEC-based completion times (from top to bottom: 1 = fast, 2 = medium, and 3 = slow) have been investigated.

All robotic action parameters (trajectories and speed) had been predefined for the five different individual actions using dynamic movement primitives (DMPs^34,35^) to encode the motion from source to target. Hence, robots were not allowed to speed up or slow down their performance, because we are here interested in the predictive gain and not in any other performance characteristic. Timings for the robot execution times of any individual action had been taken from the average human completion times (Table 2). Still, some differences in action completion time will naturally arise due to different source-target distances. Furthermore, robots are about 15–20 s slower than the humans (Table 3*, top*), for completing an action sequence, because of the quite slow speed of the robot hand for grasping and other hand-shape changes (see robot video in the Supplementary Material).

Table 3 compares average human performance (left part of the table) with robot performance on these three sequencings. Differences in sequencing times arise from the fact that some sequences allow for more efficient predictive chaining than others. A small font for human SEC performance is chosen in Table 3 to indicate that this performance would never exist in reality.

The right side of the table shows how fast the two KUKA robots can perform these sequences. As expected, ESEC-based execution is faster for humans as well as robots than SEC-based execution. Furthermore note that the ordering of 1,2,3 = fast,medium,slow only holds for average human-ESEC-based execution times. Scene geometry as well as the fact that SECs have little predictive power lead to the situation that sequence completion times do not keep this ordering for all other cases. The median values of the ESEC predictive power of the here-chosen five *individual* actions range between ***P ***= 13.1 and ***P ***= 46.0 (Fig. 3) with an average of ***P ***= 28.7. The average predictive power values of the *sequencings* investigated in Table 3 are with ***P ***= 35.9 (human) and ***P ***= 36.7 (robot) larger than the ***P ***= 28.7 average value above, because time-savings from the predictions add up to some degree. Remarkably this gain is not terrific, because many cases exist where in an action chain an agent can actually not make much use of an early prediction, for example when it has not yet finished its own action (see e.g. the time line in Fig. 4*, top*). Notably, predictive power values for human and robots are quite similar. The actual differences in individual execution times average out and the “gain” is for both types of agent comparable.

## Discussion

**Figure 5:**
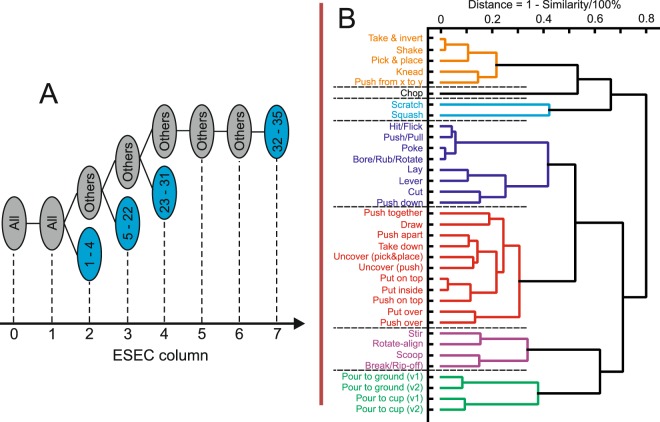
**(A**) Event columns where certain actions from an initial set of 35 actions are unequivocally recognized. For example, already at column 2, actions (1) Hit/Flick, (2) Poke, (3) Bore/Rub/Rotate, and (4) Lay will be known. The other actions are: (5) Push/Pull; (6) Stir; (7) Knead; (8) Lever; (9) Push from x to y; (10) Take & invert; (11) Shake; (12) Rotate-align; (13) Pick & place; (14) Pour from a container onto the ground when the liquid first un-touches the container then touches the ground (Pour to ground [v1]); (15) Pour from a container on the ground when the liquid can touch the container and the ground at the same time (Pour to ground [v2]); (16) Pour from a container to another container when the liquid first un-touches the container then touches another container (Pour to cup [v1]); (17) Pour from a container to another container when the liquid can touch the container and another container at the same time (Pour to cup [v2]); (18) Cut; (19) Chop; (20) Scratch; (21) Squash; (22) Draw; (23) Scoop; (24) Take down; (25) Push down; (26) Push apart; (27) Break/Rip-off; (28) Uncover by pick & place; (29) Uncover by push; (30) Put on top; (31) Put inside; (32) Push on top; (33) Push together; (34) Put over; (35) Push over. **(B)** Similarity dendrogram clustering actions with a threshold of 0.5.

The framework of ESECs had been derived from older approaches that emphasize the “grammatical” structure of human actions^17,20,25–29^. ESECs carry substantial predictive power. Figure 5 A shows at which event column different actions can be predicted, when considering a total of 35 actions (Figure replotted from figure 9 in^31^). Column 7 is the latest moment and this corresponds, on average, to less than half of a complete ESEC. In the same older study, we had observed on different real data sets that the average predictive power (***P***-value) for ESECs is above 60 as compared to a standard Hidden Markov Model-based prediction with ***P*** < 35. Furthermore, when calculating a similarity tree diagram (Fig. 5B, replotted from figure 6 in^31^) one can see that ESECs group actions roughly in the same way as humans would (with few exceptions). Hence, it appears that ESECs represent essentially a human-like action semantics. This had prompted us to ask in the current study whether humans and ESECs “preform in the same way” when predicting actions. The central finding of this study is that for most of the tested actions, indeed humans and ESECs use the same event configuration (column) for recognition. Humans take a little longer, though (blue dots in Fig. 3 are lower than the ESEC-bars). This is possibly because humans accumulate a bit more evidence until they make a prediction than ESEC. For example, a cutting action will be predicted by the ESEC at the very first millisecond where the back-and-forth cutting movement starts; humans take a bit longer until they can be sure that one cube really emulates a knife and performs cutting.

Another important aspect of the ESEC framework is that it neglects the fine timing of the individual action phases. At first, this appears problematic, because one loses finer distinctions between dynamic actions. Here we would, however, argue that most often action dynamics play a clear role in distinguishing between skilled versus less-skilled performance (e.g. a tennis expert versus a beginner), while dynamics are less relevant for action recognition and prediction per se.

On the contrary, losing time-information is a feature that helps grouping actions together in the right way. The individual action timings Table 2 show substantial standard deviations and the ESEC-based prediction distribution (red curve in Fig. 4) shows that action-sequence timings can vary widely, too. Thus, time may rather confuse than help recognition and prediction. Furthermore, in our example robotic experiments we found that the ESEC-based predictive power values for humans and robots accumulated over five actions differ only by 0.8 (human: ***P*** = 35.9, robot: ***P*** = 36.7) in spite of the fact that the robots performed the sequence much slower than humans would have. This finding is interesting, too. If robots use the events in the ESECs to predict actions then their interaction with a human should be smooth and “cognitively seamless” in spite of different action speeds, because – under these experimental conditions – humans appear to use the same (or temporally close) events for their own action prediction.

The other important aspect that is addressed by these findings concerns the bootstrapping problem that infants encounter when trying to understand objects. The number of objects exceeds the number of possible action-(types) by far. The grammatical structure of action events, provides a small and solid scaffold for recognition and the function of unknown objects can thereby more easily be deduced. Clearly, at some stage, object information will begin to play a major role. At a dinner table, picking up a knife will carry a different meaning from picking up a fork, where our framework would just state that both were picking-up actions. Still, taken together we would argue that a grammatical view onto actions may underlie human action understanding and that this type of encoding might help our cognitive development, while at the same time is should be helpful for robots especially when having to operate together with us.

## Methods

### Setup and procedures for the VR experiments

#### Ethical considerations

Experiments have been performed with 49 human participants. These experiments are not harmful and no sensitive data had been recorded and experimental data has been treated anonymously and only the instructions explained below had been given to the participants. All participants provided their informed consent to the experiments after we had explained to them the purpose and the procedure of the experiments. Experiments were performed in accordance with the ethical standards laid down by the 1964 Declaration of Helsinki. We followed the relevant guidelines of the Germany Psychological Society according to which these experiments, given the conditions explained above, do not need explicit approval by an Ethics Committee (Document: 205 28.09.2004 DPG:“Revision der auf die Forschung bezogenen ethischen Richtlinien”).

Procedures. We have used a Vive VR headset and controller released by HTC in April 2016 which features a resolution of 1080 × 1200 per eye. The main advantage of that over competing headsets is its “room scale” system, which allows for precise 3D motion tracking between two infrared base stations. This provides the opportunity to record and review actions for the experiment on a larger scale of up to 5 meters diagonally. Thus, using human demonstration of each individual action we have implemented ten actions: Hide, Cut, Chop, Take down, Put on top, Shake, Lay, Push, Uncover and Stir using differently colored blocks, where only the “Hand” was always red. Human demonstration results in jerk-free trajectories of the different moved blocks and actions look natural. For each action type, 30 different variants with different geometrical configurations and different numbers of distractors have been recorded (see Suppl. Material for example VR-videos). We performed experiments with 49 participants (m/f ratio: 34/15, age range 20–68 y, avg. 31.5 y) showing them these 300 actions in random order. Before starting, we showed 10 actions to each participant for training, where the selection panel was highlighting the shown action in green (Fig. 1A). In the actual experiments – some frames for the Hide action are shown in panel C – subjects had to press a button on the controller at the moment when they believed that they had recognized the action (Fig. 1B1). After button-press, the scene disappeared to avoid post-hoc recognition and the subjects could, without time pressure, use a pointer (Fig. 1B2) to indicate, which action they recognized.

The frames shown in Fig. 1*, C* correspond to the columns in the ESEC on the left side. At C1 the Hand is above the ground and at C2 it touches Object 1 (yellow field). Through this several other relations come now also into being (other entries at C2). Object 1 leaves the ground at C3 and at C4 the yellow field shows that it is registered as “touching” Object 2 (see note on the simulated vision process above). This leads to the emergence of many static and dynamic spatial relations (other entries in C4) and this is the column where the ESEC will unequivocally know that this is a Hide action. From there on, several more changes happen (C5–C7) until the action ends.

### Similarity measure for ESEC comparison

Note that all actions shown in the VR experiments had been generated by capturing their movement trajectories from human demonstration. In addition, different geometrical arrangements of objects and distractors were used. As a consequence, ESECs from the same action class are normally quite similar but often not identical. (Obviously, this statement is also true for ESECs that are extracted from observing human actions directly.)

Thus, we need a method to calculate the similarity of two manipulation actions. In general, different measures would be possible and the one used here (described below) had been chosen, because it discriminates all 35 actions, while keeping their sematic similarities (main text Fig. 5B) in a way similar to human general judgment.

Different from SECs, where only one type of relation exist, here we have to deal with three types, which can occur concurrently: touching/non-touching, static spatial relations (SSR) and dynamic spatial relations (DSR).

We proceed as follows: Suppose α1 and α2 are two actions. Their ESEC matrices have n and m columns, respectively. Instead of considering their 30-row ESECs individually, we concatenate the corresponding T/N, SSR and DSR of each fundamental object pair into a vector with three components and now create a 10-row matrix for α1 and α2. We annotate α1 with components x and α2 with components y. Thus, α1 becomes:

$$\alpha 1=(\begin{array}{c}({x}_{1,1},\,{x}_{11,1},\,{x}_{21,1})\,({x}_{1,2},\,{x}_{11,2},\,{x}_{21,2})\ldots \,({x}_{1,n},\,{x}_{11,n},\,{x}_{21,n})\\ \begin{array}{c}({x}_{2,1},\,{x}_{12,1},\,{x}_{22,1})\,({x}_{2,2},\,{x}_{12,2},\,{x}_{22,2})\ldots \,({x}_{2,n},\,{x}_{12,n},\,{x}_{21,n})\\ \ldots \\ ({x}_{10,1},\,{x}_{20,1},\,{x}_{30,1})\,({x}_{10,2},\,{x}_{20,2},\,{x}_{30,2})\ldots \,({x}_{10,n},\,{x}_{20,n},\,{x}_{30,n})\end{array}\end{array})$$

With these components of both matrices we define the differences in the three different relationship categories ${D}^{1:3}$ by:

$$\begin{array}{l}{D}_{i,j}^{1}=\{\begin{array}{c}0,\,\,\,\,if\,{x}_{i,j}={y}_{i,j}\\ 1,\,\,\,\,\,\,\,otherwise\end{array}\\ {D}_{i,j}^{2}=\{\begin{array}{c}0,\,\,\,\,\,if\,{x}_{i+10,j}=\,{y}_{i+10,j}\\ 1,\,\,\,\,\,\,\,\,otherwise\end{array}\\ {D}_{i,j}^{3}=\{\begin{array}{c}0,\,\,\,\,if\,{x}_{i+20,j}=\,{y}_{i+20,j}\\ 1,\,\,\,\,\,\,\,\,\,otherwise\end{array}\end{array}$$

where 1 ≤ i ≤ 10, 1 ≤ j ≤ P, P = max(n, m)

Then we define the composite difference for the three categories as follows:

$$dif{f}_{i,j}=\sqrt{{D}_{i,j}^{1}+{D}_{i,j}^{2}+{D}_{i,j}^{3}}$$

If one matrix has more columns than the other matrix, i.e. m <n or vice versa, we repeat the last column of the smaller matrix until getting the same number of columns as the bigger matrix. This leads to a consistent drop in similarity regardless of which two action are being compared. Now *Difference* is defined as a matrix, where its components represent the difference values between the two ESEC s’ corresponding components.

$$Differenc{e}_{(10,p)=}(\begin{array}{c}\begin{array}{c}dif{f}_{1,1}dif{f}_{1,2}\ldots \,dif{f}_{1,p}\\ dif{f}_{2,1}dif{f}_{2,2}\ldots \,dif{f}_{2,p}\end{array}\\ \begin{array}{c}\ldots \\ dif{f}_{10,1}dif{f}_{10,2}\ldots \,dif{f}_{10,p}\end{array}\end{array})$$

where *diff*_*i,j*_ denotes the dissimilarity of the i_th_ objects pair at the j_th_ time stamp (column). Then, *Dis*, which is the total dissimilarity between ESECs of α1 and α2 is obtained as the average across all components of the matrix *Difference*.

$$Di{s}_{\alpha 1,\alpha 2}=\frac{1}{p\,\ast \,10}(\mathop{\sum }\limits_{j=1}^{p}\mathop{\sum }\limits_{i=1}^{10}dif{f}_{i,j})$$

Accordingly, Sim_α1,α2_, the similarity between the ESECs matrices of actions α1 and α2, is obtained as the complimentary value of *Dis*.

$$Si{m}_{\alpha 1,\alpha 2}=(1-\,Di{s}_{\alpha 1,\alpha 2})\,\ast \,100$$

This is, thus, given in percent and used to quantify the similarity between any two actions in this study.

### Action prediction and quantification measure

The variability of the ESECs within any given action class requires using a probabilistic method to assess at what time point any action would be predictable by its ESEC. This is a two step procedure.

1. First, we determine the self-similarity within any given action class α_i_. This is needed to define the actions’ individual thresholds Θ_i_ for determining class ownership in a correct manner.
2. After this is done, we can measure the predictive power P. For this, we sequentially and cumulatively compare – as the columns arrive one after the other – a new action’s ESEC to the ESECs of all action classes eliminating them one-by-one until only one action remains for which we determine P.

Step 1: To account for the variability within the action videos for one given class, we measured self-similarity by pairwise computing similarity *Sim* for m = 20 randomly selected ESECs from the given action class α_i_, getting us ½m(m − 1) = 190 values. We repeat this for 20 such selections and average the resulting 3800 values. This, somewhat overly complex, procedure was used to be independent of the total number of action videos (here 30 per action class) and to allow using it in a similar way in the future when observing humans during real actions, too. After averaging, we use the resulting average *Sim* value as the similarity threshold, Θ_i_, for this action class. It is important to note that the use of just a single threshold for all action classes is incorrect, because classes have different degrees of self-similarity.

Step 2: The algorithm for computing P is as follows. We call, as above, our action classes α_1_, α_2_,…, α_10_, the columns in an ESEC C0, C1, C2,…., Cn, and the ESEC of the to-be-considered new action γ. Then we randomly select from each class α_i_ again 20 comparison actions (indexed with *j*). Note that column C0 is a “pre-set” start column and is identical for all actions. Thus, we start with column C1 and calculate the similarity values of the first column of γ to all the first columns of the selected comparison actions resulting in 20 values for *Sim*_*j*_*(C1*)_α*i*,γ_, (*j* = *1,…,20*), for C1 for all classes α_i_. These values will then be class-wise averaged across the 20 comparison actions:

$$\overline{Sim{(C1)}_{\alpha i,\gamma }}=\frac{1}{20}\mathop{\sum }\limits_{j=1}^{20}Si{m}_{j}{(C1)}_{\alpha i,\gamma }$$

In the next step, we compare these averages for all classes and sort out all classes for which the average similarity has dropped below their belonging thresholds Θ_i_ (from step 1). Then we repeat all steps using columns C1 *and* C2 and continue this way with the growing of the ESEC until eventually more and more classes drop out, up to the moment where only one class remains, which is the one to which γ belongs.

ESEC γ corresponds to a certain action video and, thus, we know the time-mapping between the movie frames and every column in the ESEC. Hence, we can calculate the moment at which the prediction happens, which is called *prediction moment T(γ*). Based on that, prediction power *P* is defined as:

$${\boldsymbol{P}}=(1-\frac{T(\gamma )}{Tot(\gamma )})\,\ast \,100 \% ,$$

where *Tot(γ)*, is the total time of the corresponding action video. The moment where the hand appears in the scene and leaves the scene are considered as the initial and last moment, respectively.

## Supplementary information

Supplementary information

Supplementary information2

Supplementary information3
