# Supplementary material for: Humans Predict Action using Grammar-like Structures
Source: Sci Rep. 2020 Mar 4;10:3999. doi: 10.1038/s41598-020-60923-5 (PMC7055263; doi:10.1038/s41598-020-60923-5)
Supplement: Supplementary file 1 — Supplementary information [file 41598_2020_60923_MOESM1_ESM.docx]

# Humans Predict Action using Grammar-like Structures

**F. Wörgötter^[[1]](#footnote-1)^, F. Ziaeetabar^1^, S. Pfeiffer^1^, O. Kaya^1^, T. Kulvicius^1^, M. Tamosiunaite^1,2^,**

^1^Universität Göttingen, Department for Computational Neuroscience at the Bernstein Center Göttingen, Inst. of Physics 3 and Leibniz Science Campus for Primate Cognition, Göttingen, Germany.

^2^Vytautas Magnus University, Faculty of Informatics, Kaunas, Lithuania.

**Supplementary Materials and Methods**

**ESECs**

The core of our work relies on the Enriched Semantic Event Chain (ESEC) framework. The main concept had been described briefly in the main text and here we summarize it again and add all missing details. An ESEC is a table consisting of three sets of 10 rows each (main text figure 1). The first set encodes the changes of the touching (T) and non-touching (N) relations for each pair of objects during a manipulation. The second set captures static spatial relations (SSR) and the third set dynamic spatial relations (DSR) between the objects. Hence, a new column is created whenever a change in any of these relations occurs. Objects are labeled as H, 1, 2, 3, and G, where H and G are “Hand” and “Ground” and Objects 1, 2, and 3 are numbered by their occurrence during the action as explained in the main text above. Actual object names are not relevant for ESEC encoding, because this encoding relies only on the sequentiallity of the different object-object relation changes. Defining ESECs with this kind of strict order of object-appearance leads to always the same row ordering in the ESEC matrix.

In the following we will describe, how the manipulated objects are modeled and – according to these models – how static and dynamic spatial object-object relations are defined.

**Object Relations**

**Touching/non-touching and other general relations:** We interpret touching/non-touching (T versus N) as the occurrence or non-occurrence of collisions between point clouds based on the k-d tree algorithm. Moreover, there are some other possible types of simple relations between objects in our framework.


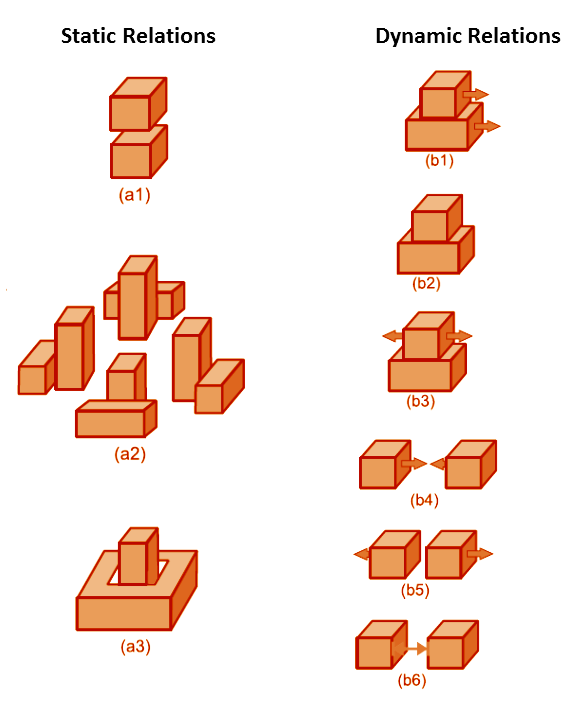


*Figure 1:* *(a) Static Spatial Relations: (a1) Above/Below, (a2) Around, (a3) Inside/Surround. (b) Dynamic Spatial Relations: (b1) Moving Together, (b2) Halting Together, (b3) Fixed-Moving Together, (b4) Getting Close, (b5) Moving Apart, (b6) Stable.*

- Undefined or “U” when an object does not exist.
- Destroyed or “X” when an object is destroyed or loses its primary shape (e.g. in cut, chop, scoop, or break actions).
- Absent or “A” when an object has already been there, but has now disappeared (e.g. in a hide action).

**Static and dynamic object relations**

Point cloud collisions using the k-d tree algorithm will not suffice to define static and/or dynamic object relations. For this, a different treatment of the objects is needed.

**Object modeling for static and dynamic object relations**

Hence, we are approximating each object by an Axis Aligned Bounding Box (AABB). In this model, all coordinate axes are aligned according to the direction of the simulated camera axes. Note that the simulated camera is fixed and does not move during the manipulations. All relations have been then defined relative to this setting.

The x axis corresponds to the right/left relation, while y and z axes define the directions of the above/below and the front/back relations, respectively.

An AABB models a point cloud by a cube with sides parallel to the directions of the coordinate system axes. AABB computation details are discussed in [1].

***Static Spatial Relations*** depend on the relative position of two objects in space. These types of relations are computed at every frame and there is no need for data from previous frames.

We define the following types of SSRs: ‘‘Above’’ (**Ab**), ‘‘Below’’ (**Be**), ‘‘Right’’ (**R**), ‘‘Left’’ (**L**), ‘‘Front’’ (**F**), ‘‘Back’’ (**Ba**), ‘‘Inside’’ (**In**), ‘‘Surround’’ (**Sa**) and ‘‘Between’’ (**Bw**). Right, Left, Front and Back are composed into ‘‘Around’’ (**AR**) and used at times when one object is surrounded by the other. Moreover, ‘‘Above’’, ‘‘Below’’ and ‘‘Around’’ relations can be combined with the “touching” relation and are then converted to “Top” (**To**), “Bottom” (**Bo**) and “Around with touch” (**ArT**), respectively. *Figure 1 (a1-a3)* represents static relations between two objects in term of cubes. Note that this way this type of encoding contains a bit of redundancy for better human readability of these tables.

If the distance between two objects’ AABBs exceeds a certain threshold, they do not have any of the above mentioned relations and their static relation is assumed as Null (**O**). Therefore, the set of static spatial relations is given by: SSR = {Ab, Be, R, L, F, Ba, Ar, To, Bo, ArT, In, Sa, Bw, O}.

Each relation is defined by a set of rules.

In general, xmin, xmax, ymin, ymax, zmin and zmax are the minimum and maximum values between the points of the object ϴ’s AABB of at the i_th_ frame along the x, y and z-axes, respectively.

Let us consider the relation ‘‘Above’’: SSR(ϴ_i_, ϴ_j_) = Ab (object ϴ_i_ is above the object ϴ_j_) if ymin(ϴ_i_) < ymin(ϴ_j_) and ymax(ϴ_i_) < ymax(ϴ_j_) as well as all the following (exception) conditions are not true: xmin(ϴ_i_) > xmax(ϴ_j_) and xmax(ϴ_i_) < xmin(ϴ_j_); zmin(ϴ_i_) > zmax(ϴ_j_) and zmax(ϴ_i_) < zmin(ϴ_j_);

The exception conditions exclude from the relation ‘‘Above’’ those cases when two AABBs do not overlap in right/left (x direction) or front/back (z direction). Several examples of objects holding relation SSR(red, blue) = Ab are shown in *Figure 2*, when the size and shift in x direction varies.

SSR(ϴ_i_, ϴ_j_) = Be (below) is defined by ymin(ϴ_i_) > ymin(ϴ_j_) and ymax(ϴ_i_) > ymax(ϴ_j_) and the same set of exception conditions. The relations R, L, F, Ba are defined in a similar way. For R and L the emphasis is on the ‘‘x’’ dimension, while for the F, Ba the emphasis is on the ‘‘z’’ dimension. For the relation ‘‘inside’’, SSR(ϴ_i_, ϴ_j_) = In, x and z coordinates of AABB ϴ_i_ must be between the x and z coordinates of AABB ϴ_j_ respectively while ymin(ϴ_j_) < ymax(ϴ_i_) ≤ ymax(ϴ_j_). Surround (Sa) relation needs opposite conditions.

For the relation “in between” (Bw), we first define the “between space” for two objects, which is given by extending the AABBs from two non-interacting objects toward each other along the camera axis and considering the intersection of these extensions. Whenever the AABB of a third object remains completely in the “between space” of the two other objects, it is assumed that the third object is “in between” (Bw) of them. The rules relating to this relation are defined by SSR (ϴ_i_, ϴ_k_, ϴ_j_) = Bw (object ϴ_k_ is between the objects ϴ_i_ and ϴ_j_): xmin(ϴ_k_) ≥ mini(xmax(ϴ_i_), xmax(ϴ_j_)) and xmax(ϴ_k_) ≤ max(xmin(ϴ_i_), xmin(ϴ_j_)) and ymin(ϴ_k_) ≥ max(ymin(ϴ_i_), xmin(ϴ_j_)) and ymax(ϴ_k_) ≤ min(ymax(ϴ_i_), xmax(ϴ_j_)) and zmin(ϴ_k_) ≥ max(zmin(ϴ_i_), xmin(ϴ_j_)) and zmax(ϴ_k_) ≤ min(zmax(ϴ_i_), xmax(ϴ_j_)).

There could be more than one static spatial relation between two objects, e.g. one object’s AABB can be both to the left and in the back of the other object’s AABB. However, we disallow this and define only one relation per object pair.


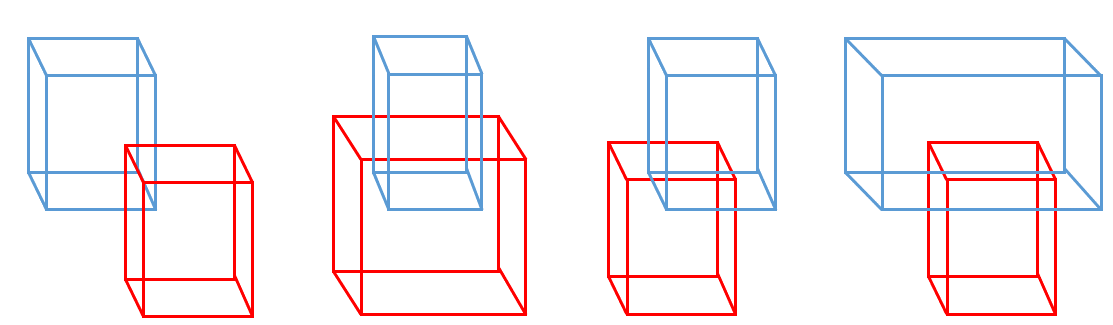


*Figure 2: Possible states of Above-Below relations between two AABBs when size and x positions vary.*

To achieve this the following procedure is adopted. Each AABB is a box, which includes six rectangular surfaces. We label them as top, bottom, right, left, front and back based on their positions in our scene coordinate system. When object ϴ_i_ is to the left of object ϴ_j_, one can make a projection from the right surface of object ϴ_i_ onto the left rectangle of object ϴ_i_ and consider only the rectangle intersection area. We call this area the “shadow”.

Suppose SSR(ϴ_i_, ϴ_j_) = {R_1_, . . . , R_k_} whith R_1_, . . . , R_k_ ∈ SSR. We calculate the *shadow*(ϴ_i_, ϴ_j_, R_m_) (1 ≤m≤k) for all relations R_m_ between objects ϴ_i_ and ϴ_j_. Then we select the relation with the biggest shadow as the main static relation between the two objects, SSR(ϴ_i_, ϴ_j_) =R_n_(1 ≤n ≤ k), if: shadow(ϴ_i_, ϴ_j_, R_n_) = max_1≤m≤k_ (Shadow(ϴ_i_, ϴ_j_, R_n_)).

Several of these static relations are dependent on the viewpoint and the exact relation is often not relevant (also humans do not consider this many times). For instance, when picking up a spoon to stir a cup of tea, it is usually not important that the spoon is picked up from the right or the left side of the cup. Thus, we define in addition a relation called ‘‘Around’’ (Ar) and gather all relations L, R, F, Ba into it. This way, ‘‘Ar’’ (Around) contains the space located *lateral* to the object in a limited radius equal to a threshold π. This space does not cover vertical neighborhoods like ‘‘Above’’ or ‘‘Below’’ [1].

***Dynamic Spatial Relations*** define the spatial relation between two objects that can be in a moving or stable condition. Here, different from SSRs, which are computable at every frame, we need some information from the previous *N* frames (e.g., distance related parameters) between each pair of objects. The parameter *N* is defined according to the frame-rate of the movie, where we determine *N* as frame count for covering 0.5 s. This has turned out to be a good heuristic estimate of the time it takes for a person to change the relations between objects. Therefore, if the video rate is μ frames per second, then *N* = 0.5μ. DSRs include the following relations: ‘‘Moving Together’’ (**MT**), ‘‘Halting Together’’ (**HT**), ‘‘Fixed-Moving Together’’ (**FMT**), ‘‘Getting Close’’ (**GC**), ‘‘Moving Apart’’ (**MA**) and ‘‘Stable’’ (**S**). These dynamic spatial relations between two objects are shown in *Figure 1 (b1–b6)* by using cubes. MT, HT and FMT define situations when two objects are touching each other while: both of them are moving in a same way (MT), are not moving (HT), or when one object is fixed and does not move, while the other one is moving on or across it (FMT). Case S (stable) denotes that any distance-change between objects is less than a defined threshold (here, we have considered this threshold as Ƹ = 1 cm) and remains constant during the action sequence. In addition, Q is used to indicate a dynamic relation between two objects if the distance between them is more than the defined threshold Ʊ = 10 cm or if they do not have any of the above defined dynamic relations. Therefore, we have defined DSR as a two argument function where the arguments are AABB cubes in the scene. Suppose c_i_^f^ shows the central point of the AABB of object e_i_^f^ (object e_i_ in f_th_ frame). For measuring the Euclidean distance between AABBs of e_i_ and e_j_ in the f_th_ frame, $\delta\left( e_{i}^{f+\partial}, e_{j}^{f+\partial} \right)=|\left| c_{i}^{f}-c_{j}^{f} \right||$ is defined by:

$$DSR\left( e_{i}^{f},e_{j}^{f} \right)=\left\{ \begin{aligned} GC if \delta\left( e_{i}^{f+\partial}, e_{j}^{f+\partial} \right)-\delta\left( e_{i}^{f},e_{j}^{f+\partial} \right)<\xi\\ MA if \delta\left( e_{i}^{f+\partial}, e_{j}^{f+\partial} \right)-\delta\left( e_{i}^{f},e_{j}^{f+\partial} \right)>\xi\end{aligned} \right.$$

We use a time window $\partial$=10 frames in our experiments (recording speed is 20 fps); the threshold $\xi$ is kept at 10 cm.

When calculating dynamic relations, we are also checking the touching relations between those two objects. For this we first define TNR, as a two argument function that illustrates whether two objects are touching or not-touching each other. This function is then used below to define several conditions:

$$\boldsymbol{Con}\boldsymbol{1:}TNR \left( e_{i}^{f}, e_{j}^{f} \right)=T \&\& TNR \left( e_{i}^{f+\partial}, e_{j}^{f+\partial} \right)=T$$

$$\boldsymbol{Con}\boldsymbol{2:}TNR \left( e_{i}^{f}, e_{j}^{f} \right)=N \&\& TNR \left( e_{i}^{f+\partial}, e_{j}^{f+\partial} \right)=N$$

$$\boldsymbol{Con}\boldsymbol{3:}c_{i}^{f}\neq c_{i}^{f+\partial}$$

$$\boldsymbol{Con}\boldsymbol{4:}c_{j}^{f}\neq c_{j}^{f+\partial}$$

$$\boldsymbol{Con}\boldsymbol{5:}\delta\left( e_{i}^{f+\partial}, e_{j}^{f+\partial} \right)-\delta\left( e_{i}^{f},e_{j}^{f+\partial} \right)<\xi$$

Now, the dynamic relations MT, HT, FMT and S are defined based on the above condition in the following way:

***MT,*** *if Con1 && Con3 && Con4*

***HT,*** *if Con1 && ~Con3 && ~Con4*

***FMT,*** *if Con1 && (Con3 XOR Con4)*

***S,*** *if Con2 && Con5*

$$DSR\left( e_{i}^{f},e_{j}^{f} \right)=$$

**Event Detection**

When performing action recognition by our artificial systems, all *object-object relations* are solely determined by vision similar to what a human observer would have to rely on. To arrive at a rigorous relational framework, object relations are determined from their bounding boxes (see Suppl. Material for details). This leads, for example, to the effect that the covering (Hide action) of an object by a cup is for the ESEC considered as a touching event when the cup is enclosing the object. In the VR experiments, which we will present below, all events are of course *a priori* known, but to make this consistent with the above described “free-observation” of an action, the same detection method has been emulated here too (“simulated vision”).

**Statistical Evaluation of Human Action Chaining**

The distributions in figure 4 in the main text were calculated by randomly choosing one out of the 30 existing realizations for each of the five actions and repeating this 10000 times, this way creating a base set. For each sample in the base set, all possible 120 permutations were then analyzed creating a total of 1,200,000 cases. For every case, the sequence completion (execution) duration was calculated when performing action prediction either using SECs or ESECs and added to the histogram. All VR setups represent realistic geometrical configurations. Hence, the resulting, histograms from more than 1 million piece-wise combined actions appear trustworthy for human action sequencing of the here-chosen five actions.

**Robotic Experiments, Experimental setup**

Our robotic setup consists of two KUKA LWR robotic arms with Schunk three-finger hands (SDH2) and an ASUS-Xtion RGB-D sensor. The scene consisted of three smaller and four bigger wooden boxes, where two different initial configurations were used (*Figure 3*). Bottom parts of the bigger boxes were removed to make hiding actions possible. The following three action sequences were performed: 1) Take down, hide, shake, push, put on top; 2) Push, put on top, shake, hide, take down; and 3) Put on top, shake, take down, hide, push (the video is available also at the Supplementary Material). Each of these action sequences were performed by using both frameworks, i.e., SEC and ESEC (in total six experiments).

**Robotic Experiments, Action execution**

Five main actions (hide, take down, put on top, push, shake) and two supportive actions (approach and leave an object) were recorded using kinesthetic guidance and making use of our library-of-action encoding [2, 3] by which they were encoded using dynamic movement primitives (DMPs, [4, 5]). Note, that durations of motions were scaled in proportion to the path length to have constant velocity on average for all motions. All visual analyses were performed using our previously developed methods [6, 7].


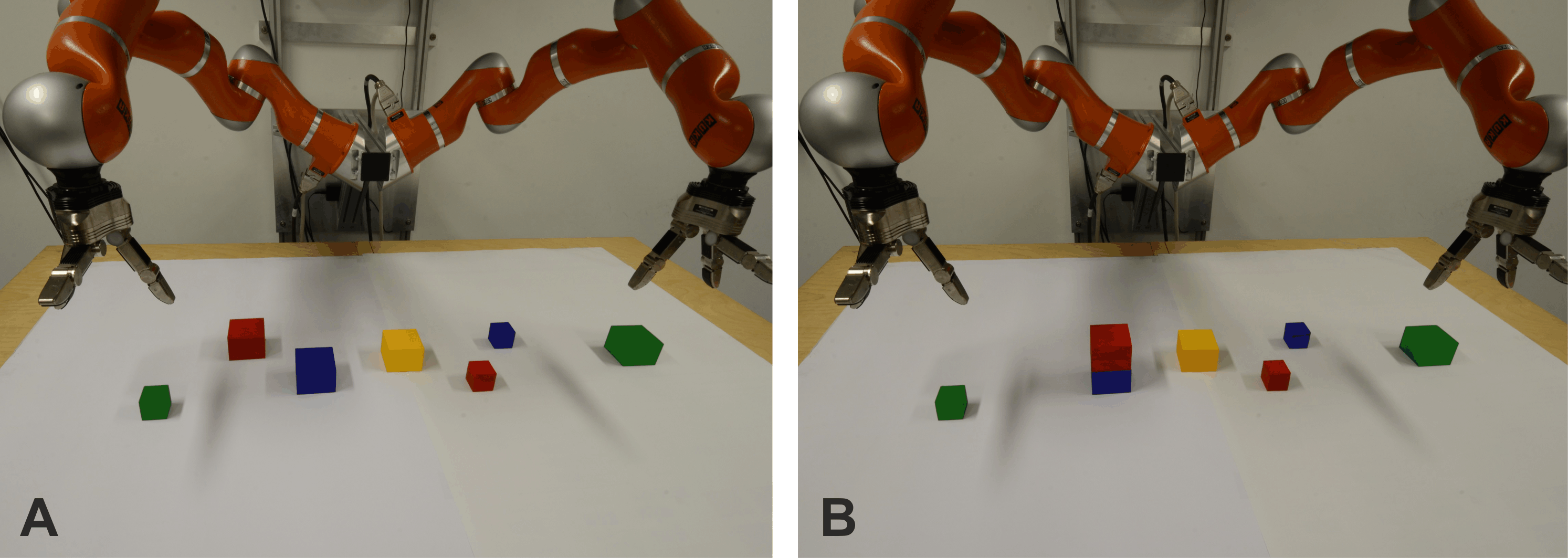


***Figure 3:*** *Two initial configurations used for robot experiments. The configuration shown on the left side was used for the action sequence 1 (take down, hide, shake, push, put on top), whereas configuration shown on the right side was used for the action sequence 2 (push, put on top, shake, hide, take down) and action sequence 3 (put on top, shake, take down, hide, push).*

References

[1] F. Ziaeetabar, T. Kulvicius, M. Tamosiunaite, and F. Wörgötter, “Recognition and prediction of manipulation actions using enriched semantic event chains,” *Robotics and Autonomous Systems*, vol. 110, pp. 173–188, 2018.

[2] M. J. Aein, E. E. Aksoy, M. Tamosiunaite, J. Papon, A. Ude, and F. Wörgötter, “Toward a library of manipulation actions based on semantic object-action relations,” in *2013 IEEE/RSJ International Conference on Intelligent Robots and Systems*, pp. 4555–4562, IEEE, 2013.

[3] M. J. Aein, E. E. Aksoy, and F. Wörgötter, “Library of actions: Implementing a generic robot execution framework by using manipulation action semantics,” *The International Journal of Robotics Research*, p. online available, 2019.

[4] A. J. Ijspeert, J. Nakanishi, and S. Schaal, “Movement imitation with nonlinear dynamical systems in humanoid robots,” in *Robotics and Automation (ICRA’02), IEEE International Conference on*, vol. 2, pp. 1398–1403, 2002.

[5] T. Kulvicius, K. Ning, M. Tamosiunaite, and F. Wörgötter, “Joining movement sequences: Modified dynamic movement primitives for robotics applications exemplified on handwriting,” *IEEE Transactions on Robotics*, vol. 28, no. 1, pp. 145–157, 2012.

[6] J. Papon, A. Abramov, M. Schoeler, and F. Worgotter, “Voxel cloud connectivity segmentation-supervoxels for point clouds,” in *Proceedings of the IEEE conference on computer vision and pattern recognition*, pp. 2027–2034, 2013.

[7] S. C. Stein, M. Schoeler, J. Papon, and F. Wörgötter, “Object partitioning using local convexity,” in *2014 IEEE Conference on Computer Vision and Pattern Recognition*, pp. 304–311, 2014.

1. * To whom correspondence should be sent at worgott@gwdg.de. [↑](#footnote-ref-1)
